# Prevalence of and factors associated with depression among adult patients living with HIV/AIDs undergoing ART unit in Banadir hospital, Mogadishu Somalia

**DOI:** 10.1186/s12888-023-04723-x

**Published:** 2023-04-07

**Authors:** Abdirahman Khalif Mohamud, Omar Abdullahi Ahmed, Abdulrahman Ahmed Mohamud, Najib Isse Dirie

**Affiliations:** 1 Faculty of Medicine and Health Science, SIMAD University, Mogadishu, Somalia; 2 Department of ART Unit, Banadir Hospital, Mogadishu, Somalia; 3 Department of Urology, Dr. Sumait Hospital, Faculty of Medicine and Health Sciences, SIMAD University, Mogadishu, Somalia

**Keywords:** HIV, AIDS, Prevalence, Factors associated, Depression, ART, Somalia

## Abstract

**Background:**

Depression is the most common psychiatric disorder in HIV/AIDs patients, and its prevalence is three times higher in HIV/AIDs patients. Globally, over 35 million people were living with HIV/AIDs, 24.7 million were in Sub-Saharan Africa. The study aims to estimate the prevalence and determine factors associated with depression among HIV/AIDs adult patients in the ART unit at Banadir Hospital Mogadishu, Somalia.

**Method:**

A hospital-based cross-sectional study was conducted between 1 May and 1 July 2022. Samples were recruited from the HIV/AIDs adult patients attending in ART unit at Banadir Hospital, Mogadishu, Somalia. A validated research tool, including sociodemographic, behavioral, clinical, and psycho-social characteristics, three items social support scale, an 11-item HIV stigma scale, and patient health questions-9 (PHQ-9) were used. The interview was conducted privet room in the ART unit. Logistic regression was used to determine factors associated with depression at the significance level a = 0.050.

**Result:**

The overall prevalence of depression among HIV/AIDs patients was 33.5% (95%CI = 28.1–39.0). In the multivariable logistic regression, three factors were associated with depression; the odds of depression were 3.415 times (95%CI=1.465–7.960) greater for those with poor social support than those with moderate-strong social support. Those with moderate and poor treatment adherence had 14.307 times (95%CI=5.361–38.182) greater odds of depression than those with good treatment adherence. Those who use substances had 3.422 times (95%CI=1.727–6.781) greater odds of having depression than those who did not.

**Conclusion:**

People living with HIV in Mogadishu, Somalia, suffer from depression. The implementation to reduce depression should be focused on empowering social support, developing an appropriate approach to increase treatment adherence, and reducing or eliminating substance use.

## Background

Depression is the most common psychiatric disorder in Human immune deficiency virus (HIV) patients [1, 2], characterized by a miserable mood, less interest or energy, feelings of fault, or low self-esteem, loss of appetite or sleep, and lack of concentration [1–4]. Globally, around 350 million people are diagnosed with depression, and it was recognized second most significant disease in 2020 [3, 4]. Low- and middle-income countries had insufficient medical care for depression, with over three-four depressed patients being misdiagnosed and cannot access treatment [3].

HIV is a viral infectious disease that affects humans and leads to vulnerability to other infections and immunological illnesses [5, 6]. Globally, over 35 million patients were living with HIV/AIDs, 24.7 million were in Sub-Saharan Africa, and about 1.6 million died from HIV/AIDs-related conditions [3, 5–7]. Almost 43% of new HIV cases in Africa were in Eastern and Southern including Somalia [7]. The prevalence of depression among the HIV-positive population is three times higher than in non-infected people. Around one in every three HIV patients suffer from depression [8–11].

The prevalence of depression among HIV/AIDs patients was 58.7% in India [12], 29.4% in Brazil [13], 54.4% in Italy [14], 37% in the USA [15], 25.4% in South Africa [16, 17], 47% in Uganda [18], and 43.9% in Ethiopia [19]. Depression in HIV patients causes health and economic impacts, including rapid HIV progress to AIDs, suicidal attempts, high treatment costs, despair, poor ART Adherence, brain damage, ART treatment failure, and poor quality of life [12]. Furthermore, HIV-depressed people may associate terrible behaviors, including engaging in unprotected sex, which is a greater risk of HIV spread [16].

Depression among the HIV-positive had several socio-demographical, psychosocial, and clinical determinants such as stigma, poor social support, anxiety, hard life, ART side effects, compromised immune status (CD4 level), presence of opportunity infections, illiteracy or low education level, financial hardship, and unemployment [11, 20–24]. Furthermore, negative social beliefs and stigma of HIV are the worst contributing factors that restrict employment and life opportunities such as good income and good education which ultimately cause patients severe depression and hand-up [25]. Unfortunately, around one-half to two-thirds of depressed patients are misdiagnosis [26, 27].

There is limited data about depression among HIV patients in Somalia, especially in Mogadishu, the capital and highest populated city, due to prevailing insecurity limits and bother investigation efforts. Furthermore, a similar study has not been implemented before in Somalia, while around 43% of new African HIV cases are in East and Southern African countries, including Somalia [7]. In addition, healthcare coverage among HIV patients in Somalia is deficient, with few ART centers in the country [28]. People living with HIV/AIDs experience a generalized HIV stigma with self-isolation that restricts life opportunities, including good income and education, which may cause severe depression. They struggle to remain employed and hesitate to seek treatment due to HIV-related discrimination, poor social support, and stigma [28, 29]. Moreover, depression is misdiagnosed because of an absence of routine screening. All this evidence shows a possibility of high depression prevalence among HIV patients. Therefore, the study aims to estimate the prevalence and determine factors associated with depression among HIV/AIDs adult patients in the ART unit at Banadir Hospital Mogadishu, Somalia.

## Method

### Study design

A hospital-based cross-sectional study was conducted to estimate the prevalence and determine factors associated with depression among HIV/AIDs adult patients in the ART unit in the study hospital between 1 May to 1 July 2022.

### Study setting

The study setting is Banadir hospital in Mogadishu, Somalia which is a public, teaching, and referral hospital established in 1977 and provides healthcare services to over three million population, including migrants. In addition, the hospital administrated by the Ministry of health in the federal republic of Somalia had the largest ART center in south and central Somalia.

### Study population

The study population was all HIV/AIDs patients with evidence of HIV/AIDS infection who had a medical record file and attended the ART unit in Banadir hospital during the data collection time.

### Inclusion and exclusion criteria

All HIV/AIDs patients who had evidence of HIV/AIDS infection attended and had record files in the study hospital were included after excluding those who had incomplete files, those who were not mentally fit, not hear or verbally communicate, had severe medical conditions, and refused to sign an informed consent form.

### Sampling procedure

The sample size was calculated based on the standard formula of a cross-sectional study [30], n = Z^2^_a/2_*P*(1-P)/d^2^, Where Z^2^_a/2_ was 1.96 level of confidence, P is the estimated proportion of prevalence which was 26.0% [16], and d is the desired level of precision which was set at 0.05. The total sample study required was 331 including an additional 10% to avoid errors. A systematic sampling technique was used to select the study participants.

### Development of research tools and variables

A well-structured reliable validated questionnaire was developed from a literature review [3–5, 16, 31] and discussed by three experts. The study’s dependent variable is depression among HIV patients, measured using Nine Patient Health Questions (PHQ-9) [3, 5, 30]. The independent variables were socio-demographic characteristics (age, sex, marital status, education, occupation, income, parenting status, residential area, and accommodation status), behavioral, clinical, and psycho-social characteristics (CD4 level, HIV stigma, social support level, duration of HIV infected, opportunistic infection status, HIV stages, ART regiment line, ART adherence, HIV status disclosure, and substance abuse).

To measure social support level three-item social support scale use categorized by (3–8 “poor support,“ 9–11 “moderate support,“ and 12–14 “strong support”) [32]. ART adherence was measured by calculating the number of pills from the prescribed drugs remembered last month. Those who remembered ≥ 95% recognized good adherence, while those who remembered < 95% recognized moderate-poor adherence [33]. An 11-item of HIV Stigma scale used to measure HIV-related perceived stigma, and stigma variables were computed to build a single stigma variable classified as stigmatized and non-stigmatized by using a cutoff point of the mean of stigma variables [34, 35]. Those chewing charts, smoking cigarettes, using tobacco or alcohol, using intravenous drug abuse, and so on were recognized as substance abusers [2].

The study tool was initially developed in English, and then language experts did forward-backwards translation to verify the consistency. The study tool was validated using the item objective congruence (IOC) method [36] for content validity by three external experts (psychiatrist, infectious disease specialist, and clinical researcher). Subsequently, to ensure reliability and respondents’ understanding of the questionnaire, a 30-respondent pilot study was conducted in the study hospital, and an acceptable Cronbach’s alpha of 0.78 was achieved.

### Process of data collection

Data collectors were trained to understand the questionnaire’s content deeply. A face-to-face interview was implemented, and patient records files were reviewed in a confidential private room lasting 30 min individually, and the hospital director approved patients’ records access.

### Statistical analysis

Data were cleaned, coded, and entered on the speeded sheet imported into the SPSS version 20 (SPSS, Chicago, IL) for analysis. The general characteristics of samples were analyzed according to percentages because all data were categorical. Logistic regression in univariate and multivariate models was used to determine factors associated with depression. Variables with a p-value < 0.05 in univariate logistic regression were a candidate and included in multivariate logistic regression. The Hosmer-Lemeshow goodness of fit test was used to indicate the final model goodness of fit. In multivariate logistic regression, variables having a p-value < 0.05 was considered statistically significant.

### Ethical consideration

This study was conducted following the rules and regulations of the World Medical Association’s declaration of Helsinki. Ethical clearance was obtained from the Banadir Hospital review board. Banadir Hospital’s ethical review board approved the process with approval number: *IRB Ref no*:-*2022/02/BH0039* since it was observational research with no intervention and no adverse on the participants. All eligible respondents explained the study objectives and requested participation. Illiterate respondents explained the study objectives through legal guardians without influence or coercion on their decision. Agreed participants gave written informed consent to sign or fingerprint for illiterate participants and only agreed participants were included in the study. Moreover, all participants were informed that they had a full right to participate or discontinue the interview at any time, confidentiality was kept, questionnaires were anonymous, and data were only present as a general number without reflecting individual information.

## Results

### Sociodemographic variables

Three hundred thirty-one respondents were recruited for the study; 51.1% were female, 53.2% were married, and 68.6% were aged between 30 and 45. Almost half (47.1%) were illiterate, and 59.8% were employed. 46.2% had a monthly income of less than 100 USD, and 92.7% disclosed their HIV status (Table 1).

**Table 1:** Sociodemographic characteristics of participants

Characteristics	n	%
	331	100.0
Sex		
Female	169	51.1
Male	162	48.9
Marital status		
Married	176	53.2
Unmarried	155	46.8
Age (years)		
18–30	71	21.5
30–45	227	68.6
> 45	33	10.0
Education		
Illiterate	156	47.1
Primary	126	38.1
Post-primary	49	14.8
Occupation		
Employed	198	59.8
Jobless	133	40.2
Monthly income $US		
< 100	153	46.2
100–300	147	44.4
>300	31	9.4
Have children		
Yes	289	87.3
No	42	12.7
Residence area		
Rural	36	10.9
Urban	295	89.1
Accommodation status		
Rental	258	77.9
Owner	73	22.1
HIV status disclosure		
Yes	307	92.7
No	24	7.3

### Clinical, behavioral, and psycho-social characteristics

Two-thirds of the respondent’s CD4 level is between 200 and 1000, 74% not perceived stigma, 74.9% have moderate-Strong social support, 70.1% lived with HIV between 1 and 3 years, 80.2% did not have an opportunistic infection, 84.9% were in HIV Stage II, and 69.5% does not use substances (Table 2).

**Table 2:** Clinical, behavioral, and psycho-social characteristics

Characteristics	n	%
Total	331	100.0
CD4 level (cell/dL)		
<200	46	13.9
200–1000	219	66.2
>1000	66	19.9
Perceived stigma		
Stigmatized	86	26.0
Non-stigmatized	245	74.0
Social support		
Poor	83	25.1
Moderate- Strong	248	74.9
Years HIV Infected (years)		
< 1	47	14.2
1–3	232	70.1
>3	52	15.7
Opportunistic infection		
Yes	39	11.8
No	292	88.2
HIV stage		
Stage I/II	281	84.9
Stage III/IV	50	15.1
Treatment type		
First-line	289	87.3
s-line	42	12.7
Treatment adherence		
Good	258	77.9
Moderate-Poor	73	22.1
Substance abuse		
No	230	69.5
Yes	101	30.5
Smocking cigarette	32	9.7
Chewing chart	28	8.5
Use both cigarette and chart	40	12.1
Use other substances	1	0.3

### Prevalences of depression among PLWH

The overall prevalence of depression among adult HIV/AIDs patients was 111 (33.5%) with (95% CI = 28.1–39.0): Levels of depression were 22.4%, 10.3%, 0.6%, and 0.3% in mild, moderate, moderately severe, and severe depression respectively (Table 3).

**Table 3:** Prevalence of depression

Characteristics	n = 331	%	95%CI
Having depression			
No	220	66.5	61.0–71.9
Yes	111	33.5	28.1–39.0
Level of depression			
Mild	*74*	*22.4*	*17.8–26.9*
Moderate	*34*	*10.3*	*6.9–13.9*
Moderately Severe	*2*	*0.6*	*-*
Severe	*1*	*0.3*	*-*

### Factors associated with depression

In the Bivariate logistic analysis model, fourteen (14) factors were found to be associated with having depression: Marital status, education level, occupation, monthly income, residency area, CD4 level, stigma, social support, opportunistic infection, HIV/AIDS stage, treatment type, treatment adherence, HIV status disclosure, and substance use (Tables 4 and 5).

**Table 4:** Association of demographic characteristics and depression among an adult patient’s living with HIV/AIDs

Characteristics	Depression		OR (95%CI)	p-value	AOR (95% CI)	p-value
	Yes (%)	No (%)				
**Sex**						
Male	47(29.0)	115 (71.0)	1.00			
Female	64(37.9)	105(62.1)	1.49 (0.94–2.36)	0.089		
**Marital status**						
Unmarried	64 (41.3)	91 (58.7)	1.93(1.21–3.06)	0.005*		
Married	47 (26.7)	129 (73.3)	1.00			
**Age** (years)						
18–30	17 (23.9)	54 (76.1)	1.00			
31–45	82 (36.1)	145(63.9)	1.79 (0.97–3.30)	0.059		
≥46	12 (36.4)	21(63.6)	1.81 (0.74–4.44)	0.191		
**Education level**						
Illiterate	74(47.4)	82 (52.6)	1.00			
Primary	26(20.6)	100(79.4)	0.288(0.16–0.49)	< 0.001*		
Post-primary	11(22.4)	38(77.6)	0.321(0.153–0.672)	0.003*		
**Occupation**						
Have job	56(28.3)	142(71.7)	1.00			
Jobless	55 (41.4)	78(58.6)	1.78 (1.12–2.84)	0.014*		
**Month income** ($US)						
< 100	71 (46.4)	82 (53.6)	5.84(1.95–17.50)	0.020*		
100–300	36(24.5)	111(75.5)	2.18(0.71–6.67)	0.169		
>300	4(12.9)	27(87.1)	1.00			
**Have children**						
Yes	98(33.9)	191(66.1)	1.14 (0.56–2.30)	0.705		
No	13(31.0)	29 (69.0)	1.00			
**Residence area**						
Rural	23(63.9)	13(36.1)	4.16 (2.01–8.58)	< 0.001*		
Urban	88(29.8)	207(70.2)	1.00			
**Accommodation status**						
Rental	86(33.3)	172(66.7)	1.00			
Owner	25(34.2)	48(65.8)	1.04(0.60–1.80)	0.884		

*Significant level at α = 0.05

**Table 5:** Associations of clinical characteristics and depression among an adult patient’s living with HIV/AIDs

Characteristics	Depression		OR (95%CI)	p-value	AOR (95% CI)	p-value
	Yes (%)	No (%)				
**CD4 level** (cell/dL)						
<200	11(23.9%)	35(76.1%)	0.037 (0.013–0.105)	< 0.001*		
200–1000	41(18.7%)	178(81.3%)	0.027 (0.012–0.064)	< 0.001*		
>1000	59(89.4)	7(10.6)	1			
**Perceived stigma**						
Stigmatized	66(76.7)	20(23.3)	14.66 (8.08–26.61)	< 0.001*		
Non-stigmatized	45(18.4)	200(81.6)	1.00			
**Social support**						
Poor	65(78.3)	18(21.7)	15.85(8.59–29.25)	< 0.001*	3.415 (1.465–7.960)	0.004*
Moderate- Strong	46(18.5)	202(81.5)	1.00		1.00	
**Years HIV Infected** (years)						
< 1	16(34.0)	31(66.0)	1.00			
1–3	77(33.2)	155(66.8)	0.96(0.496–1.867)	0.910		
>3	18(34.6)	34(65.4)	1.02(0.447–2.354)	0.952		
**Having opportunistic infection currently**						
Yes	36(92.3)	3(7.7)	34.72 (10.3–116.0)	< 0.001*		
No	75(25.7)	217(74.3)	1.00			
**HIV stage**						
Stage I/ II	67(23.8)	214(76.2)	1.00			
Stage III/IV	44(88.0)	6(12.0)	23.42(9.56–57.38)	< 0.001*		
**Treatment type**						
First-line	71(24.6)	218(75.4)	1.00			
s-line	40(95.2)	2(4.8)	61.40(14.47–260.50)	< 0.001*		
**Treatment adherence**						
Good	45(17.4)	213(82.6)	1.00		1.00	
Moderate- Poor	66(90.4)	7(9.6)	44.62(19.21-103.67.55)	< 0.001*	14.307(5.361–38.182)	< 0.001*
**HIV status disclosure**						
Yes	97(31.6)	210(68.4)	1.00			
No	14(58.3)	10(41.7)	3.03 (1.30–7.06)	0.010*		
**Substance abuse**						
Yes	54(53.5)	47(46.5)	3.48 (2.13–5.70)	< 0.001*	3.422 (1.727–6.781)	0.001*
No	57(24.8)	173(75.2)	1.00		1.00	

*Significant level at α = 0.05

These variables were candidates for multivariable logistic regression, and three factors were associated with depression. The Odds of depression were 3.415 times (95%CI=1.465–7.960) greater for those with poor social support than those with moderate-strong social support. Those with Moderate-poor treatment adherence had 14.307 times (95%CI=5.361–38.182) greater odds of depression than those with good treatment adherence. Those who use substances had 3.422 times (95%CI=1.727–6.781) greater odds of having depression than those who did not (Table 5).

## Discussion

People living with HIV/AIDs in Mogadishu, Somalia, suffer from Depression, particularly those with poor social support, moderate and poor treatment adherence, and use of substances. The prevalence of Depression among HIV patients in this study is lower than the similar studies conducted in Southeast Ethiopia [2], Addis Ababa, Ethiopia [31], Debre Birham, and northern Showa in Ethiopia [37, 38], India at different times [12, 39], Brazil, Denmark, and north central Nigeria [40–42]. While is higher than in Hawassa and southwest Ethiopia [43, 44]. These variations could be related to differences in sample size, study instruments used to determine Depression, study population differences, geographical locations, and respondent beliefs. However, this study recommended early depression diagnosis and providing rotten screening for this vulnerable population.

This study revealed Odds of Depression were higher for those with poor social support than those with moderate-strong social support. Negative social behavior leads to Depression, and HIV patients avoid seeking help from others due to social stigma, which increases their loneliness, isolation, and Depression. Previous studies conducted in India, Cameroon, and two different areas in China supported this [12, 19, 45, 46].

Depression was higher for those with moderate and poor treatment adherence than for good adherence. Antiretroviral therapy (ART) is the only technique to suppress HIV without a client’s perfect adherence is hard to achieve. Perfect ART adherence is crucial to effective HIV treatment. It is vital in sustaining viral suppression, restoring immune function, reducing morbidity, mortality, and occurrence of opportunistic infections, and enhancing the quality of life. Poor ART adherence has numerous impacts, including drug resistance, high mortality rates, poor treatments outcome, and a high prevalence of opportunistic infections [47]. In addition, the average ART adherence in Sub-Saharan Africa was 72.9% [48]. A systematic review and meta-analysis in Africa reported ART adherence between 32.9 and 94% [49]. People living with HIV in Sub-Saharan Africa often default on medication due to difficulty accessing health care and being forgetful. This paper re-emphasizes improving awareness toward ART adherence, providing ART retention mechanisms, and support services among HIV-infected people. Similar studies in Addis Ababa and Jimma, Ethiopia, supported this [31, 44].

In this study, the odds of Depression were higher for those who use substances than those who do not. Drug addiction and alcoholic patients are often familiar with Depression which triggers feelings of loneliness, sadness, and hopelessness. Substance abuse and psychiatric disorders among HIV-infected people often are coexisting conditions without intervention, can cause severe mental illness, increase the risk of engaging in unprotected sex, and decrease ART adherence that, ultimately causes HIV to spread with excessive mortality. Similar studies are in line [3, 5, 50, 51].

Stigma in people living with HIV is a predisposing factor for Depression globally since it is widespread in Somalia with self-isolation that restricts life opportunities, and they struggle to remain employed due to discrimination and poor social acceptance [28, 29]. Stigma increases fatigue, decreases the feeling of worthlessness, increases the feeling of shame, fear of HIV disclosure, isolation, and despair. All these enhance Depression and may cause treatment failure. Studies done in Debir birahan, Ethiopia and Denmark supported this [52, 53].

In addition, previous literature reported some socio-demographic factors associated with Depression, but this study did not find any. Marital status had a significant in comorbidity with Depression. Unmarried HIV patients were more likely to have Depression than those with a stable marital life [31, 43, 44, 52]. Unstable marital status or loss of a partner due to HIV predisposes to Depression, and the presence of good social support can be a buffer [31]. A good education protects against Depression, while illiteracy is a risk factor [52]. Joblessness and financial hardship were also related to Depression, particularly job loss due to HIV stigma may fall to excessive problematic thinking with untimely results in mental health and Depression [52]. Finally, the opportunistic infection synergizes depression and disease progression to AIDs [38].

## Conclusion

The prevalence of Depression among HIV/AIDS patients in Mogadishu, Somalia, is high, and poor social support, poor and fir treatment adherence, and used substances were factors associated with it. Hence Depression is still undiagnosed and untreated, and further generalized research targeting a high population is needed to understand its cause and associated factors clearly. In addition, health governmental agencies, other stakeholders, and healthcare workers should emphasize early diagnosis with early response to Depression among HIV patients.

## Study Limitations

This was the first similar study done in Somalia; the first limitations were the absence of previous comparable study. In addition, the study was conducted in a hospital-based so the finding may differ from the general population.

## Data Availability

All datasets generated and analyzed during the current study are included in this article.

## Abbreviations

ART: Anti-retroviral therapy
HAART: High active anti-retroviral therapy
OIs: Opportunistic infections
PHQ-9: Patient health questions-9
HIV: Human immune deficiency virus
AIDs: Acquired immune deficiency
PLWH: Patient living with HIV
